# Open Hole Tension of 3D Printed Aligned Discontinuous Composites

**DOI:** 10.3390/ma15238698

**Published:** 2022-12-06

**Authors:** Narongkorn Krajangsawasdi, Ian Hamerton, Benjamin K. S. Woods, Dmitry S. Ivanov, Marco L. Longana

**Affiliations:** Department of Aerospace Engineering, Bristol Composites Institute, School of Civil, Aerospace, and Mechanical Engineering, University of Bristol, Queen’s Building, University Walk, Bristol BS8 1TR, UK

**Keywords:** aligned discontinuous fibre composites, thermoplastic composite, additive manufacturing, 3D printing, open-hole tensile test

## Abstract

This paper explores the use of Discontinuous Aligned Fibre Filament (DcAFF), a novel discontinuous fibre reinforced thermoplastic filament for 3D printing, to produce structural complex parts. Compared to conventional composite manufacturing, 3D printing has great potential in steering fibres around small structural features. In this current study, the initial thin carbon fibre (CF)-poly(L-lactic acid) (PLA) tape, produced with the High Performance Discontinuous Fibre (HiPerDiF) technology, is now reshaped into a circular cross-section filament, the DcAFF, using a bespoke machine designed to be scalable to high production rates rather than using a labour-intensive manual moulding method as in previous work. The filaments are then fed to a general-purpose 3D printer. Tensile and open-hole tensile tests were considered in this paper for mechanical and processability of DcAFF. The 3D printed specimens fabricated with the DcAFF show superior tensile properties compared to other PLA-based 3D printed composites, even those containing continuous fibres. Curvilinear open-hole tensile test samples were fabricated to explore the processability and performances of such material in complex shapes. The mechanical performance of the produced specimens was benchmarked against conventionally laid-up specimens with a cut hole. Although the steered specimens produced have lower strength than the fully consolidated samples, the raster generated by the printing path has turned the failure mechanism of the composite from brittle to ductile.

## 1. Introduction

Small holes act as the stress concentration points in structural parts but they are necessary for most structures as fastening points or maintenance accesses. The stress concentration around the hole can cause more serious problems in anisotropic materials, such as fibre reinforced composites, because the composites are more sensitive to damage occurring at the various material scales. Special layup or extra manufacturing techniques, e.g., patching [1] and inserts [2], are considered to reduce the stress concentration around the material subtraction at the hole. Fibre shearing technology can be exploited to obtain a curvature for a circular hole with automated fibre placement technologies [3], but this method is suitable only for large radiuses.

Fused filament forming (FFF), or 3D printing, builds parts by layer-by-layer manufacturing, increasing design freedom and allowing production of complex geometries to the near-net shape. FFF offers the ability to design the structure of the object by defining the deposition path to reduce the stress concentration and change the stress flow [4,5,6,7,8,9,10]. There are several 3D printing studies about the customized printing path to reduce the stress concentration around the hole. An optimized printing path called Curvilinear Variable Stiffness (CVS), inspired by considering fluid flow around a circular obstacle, showed a significant strength improvement compared to a unidirectional composite straight fibres printing path and a drilled hole [11]. Concentric ring printing to the hole shape is another potential option that is known to reduce stress concentration [8,12]. The combination of a concentric ring and a curvilinear path in different layers aiming at maintaining the hole shape and delaying the initial failure showed promising strength compared to only the curvilinear path around the hole [9].

Although the 3D printing of the hole shape can reduce the stress concentration, the overall mechanical properties of the common 3D printing material, thermoplastics, e.g., poly(acrylonitrile-butadiene-styrene) (ABS), poly(L-lactic acid) (PLA), or polyamides (nylon, PA), are generally poor, so FFF products are normally used as prototypes or secondary structure parts [13]. To expand the application of FFF to more structural parts, the thermoplastics used in the process need to be reinforced with fibres.

Several fibre architectures from short/micro to continuous fibre have been used as a reinforcement to the thermoplastic for 3D printing. Although the continuous fibre offers a great mechanical improvement, the low steering flexibility of the fibre creates a fibreless area during the fabrication of a small radius [14]. However, the short/micro fibre that has formability similar to neat thermoplastic shows a very minor mechanical improvement compared to the unreinforced polymer [13]. Among those fibre architectures, highly aligned short fibre or aligned discontinuous fibre composites (ADFRCs) are the most promising fibre reinforcement architecture, offering a mechanical performance comparable to that of continuous fibres while retaining the formability of short fibres [15]. A novel fibre alignment technology, called High Performance Discontinuous Fibre (HiPerDiF), invented and patented by the University of Bristol, allows the production of aligned discontinuous fibre preforms to be used to reinforce various types of polymeric matrices. The sudden momentum change of a fibre–water suspension upon the impact on the furthermost of two parallel plates is exploited to align the fibre along the gap direction; the suspension is then deposited on a conveyor mesh belt where the water is extracted to obtain a dry fibre preform [16]. The produced preform is finally impregnated with a matrix, either thermosetting, thermoplastic, or vitrimeric, to obtain a thin prepreg tape [17].

In a previous study [18], carbon fibre preforms produced with the HiPerDiF were used as a reinforcement for PLA: a thin HiPerDiF-PLA tape was re-shaped by compression in a metallic mould under high temperature and finally pultruded to form a 1.2 mm diameter circular cross section suitable to be fed to a general 3D printer. The fine circular cross-section filament offers the possibility to manufacture more complex geometries with fewer defects than flat tape. This reinforcement shows a significant improvement in the tensile properties of the 3D printed part when compared to neat PLA. Even at low fibre volume fractions, 10–18%, tensile stiffness up to 14 GPa and tensile strength up to 104 MPa could be achieved compared to 2–3 GPa stiffness and around 50 MPa for pure PLA. Specimens 3D-printed with HiPerDiF-PLA filaments showed mechanical properties comparable to those of continuous CF-reinforced PLA, demonstrating that the HiPerDiF reinforcement has the potential to strengthen the thermoplastic for FFF application while providing much greater potential in terms of fibre steering.

One of the limitations of the research presented in previous studies is that the filament-forming was performed manually [18], which is time and energy consuming and can only batch-produce a few centimetres of filament per hour. This is not sufficient for the printing of large parts. A new industrially scalable DcAFF filament forming method, designed to continuously produce HiPerDiF-PLA filament, has been developed and described in Section 2. The tensile characterisation, showing the specimen preparation and the tensile properties of the printed part compared to the post-printing thermal consolidated part in order to explore the limitation of this material, is described in Section 3. The main objective of this paper, which is the evaluation of the 3D printed open hole specimens with the DcAFF material using a general 3D printer, is detailed in Section 4. An open hole tensile testing sample will be used as a showcase for both the steering potential of the material around the tight radii during the fabrication and mechanical behaviour changing by steering filament around the hole rather than cutting fibre by a material subtraction method. The open hole 3D printed with DcAFF with an adapted printing path will be benchmarked against producing straight samples with the same material system-original width and thin HiPerDiF tape manually laid and consolidated in an oven, where an open hole is obtained by an extra machining step, i.e., punching. This comparison was conducted to show the differences between additive and subtractive manufacturing when using a material with the same microstructure to construct a similar geometry. In this early stage of the 3D printing material, the printing precision needs to be improved. Section 5 will give a suggestion on the method to improve the printing precision with a general 3D printer.

## 2. Materials and Filament Forming Method

### 2.1. Materials Properties and Preparation

The matrix chosen for this study is a commercial transparent PLA filament, commonly used in 3D printing applications, supplied by 3D4Makers B.V. To integrate it with fibres, the material was first printed to produce a thin 200 mm × 13 mm film with 0.1 mm thickness. The fibres used to produce the HiPerDiF preform are commercial 3-mm chopped C124 carbon fibre with a fibre diameter of 7 μm, supplied by Toho Tenax GmbH [19]. The fibres were suspended in water at the calculated concentration, 0.001–0.003% of the fibre in volume, and processed through HiPerDiF to obtain an aligned discontinuous fibre preform. The fibre length was retained during the HiPerDiF process, which delivered a fibre alignment comparable to previous publications [16,20]. The relevant properties of both matrix and fibre used in this study were summarised in Table 1.

### 2.2. Filament Forming Method with the Industrially Scalable Method

**Figure 1:**
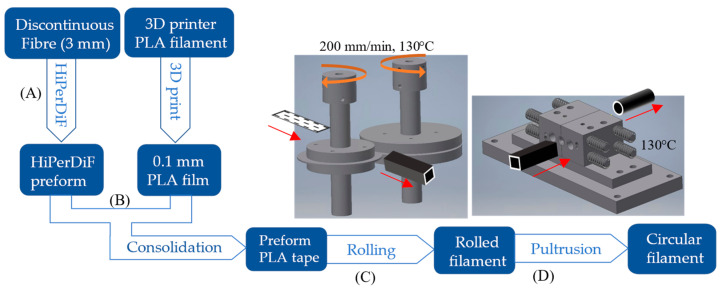
Overall DcAFF filament forming process starting from (**A**) HiPerDiF preform fibre and thin PLA matrix preparation, (**B**) consolidation of HiPerDiF preform and PLA tape, (**C**) bulking tape by designed rolling technique, and (**D**) pultrusion through a series of nozzles.

Figure 1 shows the overall process starting from the constitutive materials and ending with a pultrusion process to achieve a circular filament. The HiPerDiF preform and thin PLA 3D printed film were impregnated using heat and a compression force followed by cooling in a dedicated consolidation machine. This manufacturing arrangement and resultant sample morphology were described in detail by Krajangsawasdi et al. [17]. The initial format of the HiPerDiF-PLA in this study is a thin pre-impregnated tape with a nominal 0.2 × 5 mm^2^ cross section. To change the shape from thin tape to a circular cross section (approximately 1 mm^2^ in diameter), an intermediate step is necessary. First, the tape is transformed into a square-like cross section and then pultruded through a conical nozzle allowing it to obtain the required circular shape.

The tape was bulked by feeding it through the gap (approximately 1 × 1 mm^2^) between two heated, male and female, rollers, as shown in Figure 1 stage C. The roller moulding concept was adapted from the metal rolling process used to form metal profiles. The rollers were machined with an aluminium shaft to 50 mm in diameter with a male and a female shape at the compression point. Both rollers were heated with cartridge heaters that were inserted into the axis of the rollers. The temperature of both rollers is set just below the melting point of the HiPerDiF-PLA material, around 130 °C, to allow the material to soften and fuse, but not melt, which helps prevent tearing.

**Figure 2:**
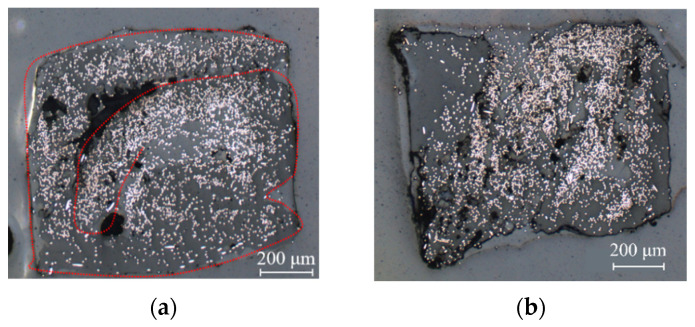

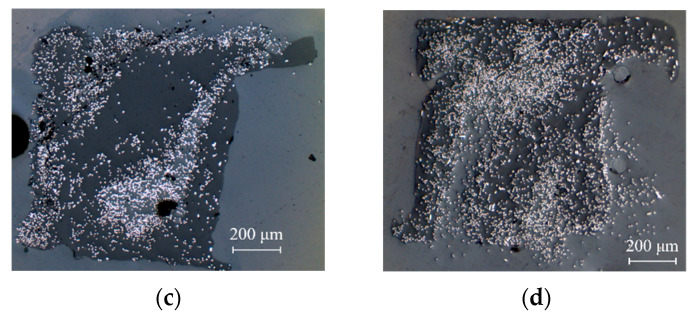
Square filament after passing through the rollers: (**a**) folding of tape with poor bonding; (**b**) microvoids in the filament; (**c**,**d**) material overflow at the corners.

The rotation of the rollers was driven by stepper motors with a controlled linear speed of 200 mm per minute. After the bulking stage, another pulling motor was used to drag the square filament away from the rollers. The linear pulling speed was synchronized to the roller speed to avoid a pulling force on the filament. Microscopic images of the cross sections of various filaments following passage through the moulding rollers are shown in Figure 2. When the gap between the rollers is too wide, the tape is folded and not well homogenised, presenting a curved shape with a large void area of imperfect fusion (see Figure 2a), which is difficult to eliminate at the next step, or some microvoids (see Figure 2b), which are more acceptable than a single large void as it can be removed by the next pultrusion. If in the initial setup, the two rollers are too close, then material overflows are produced, created by the small gaps between the male and female rollers presenting themselves as “ears” on the corners of the square shape (see Figure 2c,d). The ears are preferable to the inner voids as they can be reduced by manufacturing precise dimensions between the male and female rollers. Microvoids and “ears” imperfections, as seen in Figure 2b–d, are acceptable at this stage, as a further step allows minimising them and mitigating their effect on the filament properties.

**Figure 3:**
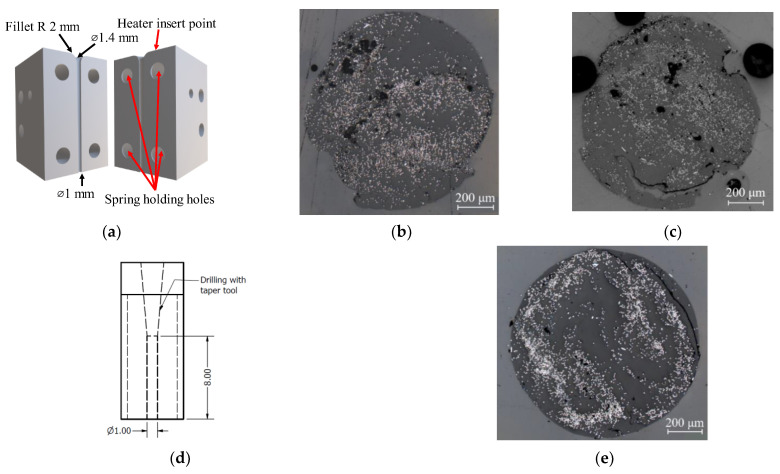
(**a**) A schematic of a two-side assembly convergent nozzle; (**b**,**c**) circular filament after passing through the two-side convergent nozzle; (**d**) the cross section of final pultrusion nozzle; (**e**) the final perfect circular cross section filament.

To shape the filament cross section into a circular shape, the square filament (with imperfections) was pultruded through a specifically designed moveable nozzle (stage D in Figure 1). The moveable nozzle was designed as two mirror parts that can be assembled into a convergent nozzle (Figure 3a) with a conical hole with a bore tapering from 1.4 mm to 1 mm. The two-side blocks are compressed with springs to ensure that they are always in contact during the process and form a circular filament while they can move slightly to allow large overflows to pass through. The nozzle was heated to 130 °C to soften the matrix and allow the reshaping of the filament in an almost circular cross-section, as shown in Figure 3b. If the material overflow is compressed, this causes an improper fusion of the matrix, as shown in Figure 3c. Both figures show an imperfect circular shape caused by the joint between two sides of the half-circle. To refine the shape and improve surface finishing, the filament was finally pultruded through a straight drilled PTFE polymer nozzle with a diameter of 1 mm and a length of 8 mm (Figure 3d) at the temperature of 130 °C. This also applies a compression force due to the thermal stress in the heated nozzle, resulting in lower voids and better surface finishing as demonstrated by the perfect circular cross sections shown in Figure 3e. The fibre distribution in the cross section cannot be controlled with this filament forming procedure leading to inhomogeneities in the filament. However, this non-uniform fibre distribution is also found in commercial 3D printing composite filaments [14,22].

### 2.3. Fibre Content Investigation in Filament

**Figure 4:**
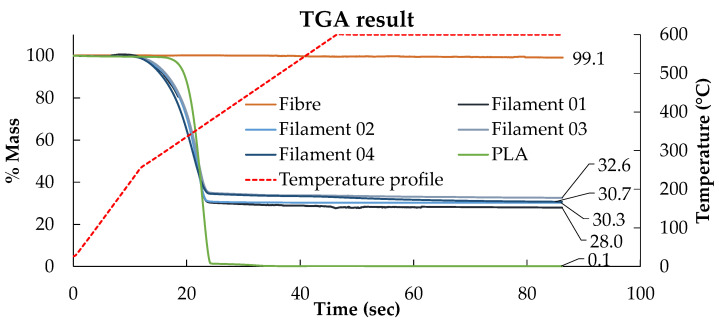
TGA programme to investigate fibre weight fraction by considering residual fibre content after matrix burn-off showing the fibre, DcAFF (HiPerDiF-PLA) composite, and PLA with the temperature profile (red line).

The fibre content of the produced filament was investigated by the matrix burn-off method with a special thermogravimetric analysis (TGA) cycle developed to calculate fibre weight fraction in composite materials [23]. The sample was heated rapidly to 250 °C with a 20 °C/min ramp followed by a 10 °C/min heating rate to 600 °C and then held isothermally for 40 min. Besides the produced HiPerDiF-PLA composite filament, the fibre preform and the printed PLA were also tested under the same programme to verify their residual mass after the dwelling stage. According to the TGA results in Figure 4, the mass losses in the fibre and the PLA residual mass after the dwelling are negligible. The residual mass percentage after the matrix burn-off procedure is the fibre weight ratio in the filament, which ranges from 28 to 33%, equivalent to a fibre volume fraction between 21 and 25%.

In a previous study [24], it was observed that in a short (<0.3 mm) randomly oriented fibre composite filament, a fibre content above 40% by weight caused nozzle clogging during printing; however, the DcAFF filament, made of highly aligned 3 mm long fibres, is expected to experience less clogging, due to the lower fibre content aided by a better alignment.

## 3. Tensile Properties Characterisation

### 3.1. Tensile Specimen Preparation

**Figure 5:**
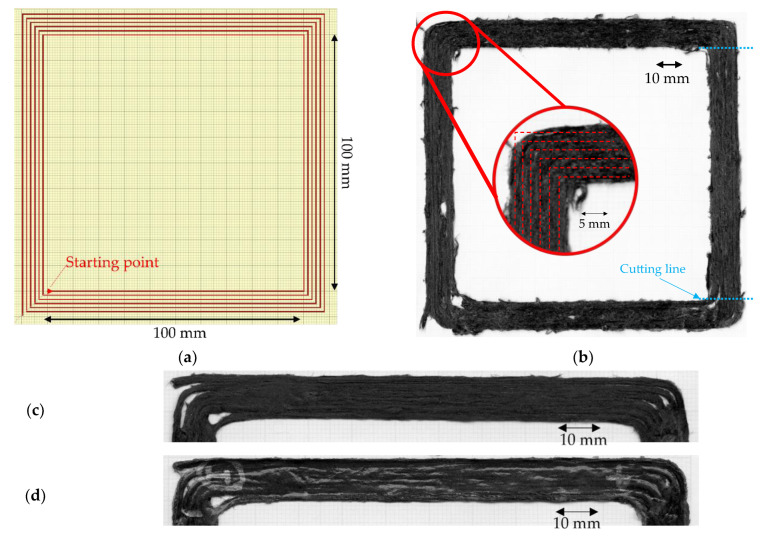
(**a**) Printing path of large square shape with 90° sharp turning corners to produce four tensile samples starting from inside; (**b**) top surface of the sharp corner printed part with a zoom-in of a corner [25]; (**c**) high magnification of the large square as printed; (**d**) high magnification of the large square printed after post-printing consolidation.

The tensile specimen is often produced using a simple 2D rectangle shape. However, for the small specimens, steering around a very tight corner is required, i.e., 90° turning, when the head reverses the print direction. This many tight turnings makes the sample difficult to repeatably produce. A new printing path for a uniaxial tensile sample to reduce the total turning corner on each printing was designed. The printing path was programmed as a single layer of 100 mm × 100 mm square spiral to produce four samples of 100 mm × 10 mm in one printing, as shown in Figure 5a. The printing was performed with an Ender3 3D printer with a 1.4-mm diameter modified flat brass nozzle. The printing set-up conditions, defined in a previous paper [18], were: nozzle temperature 210 °C, bed temperature 80 °C, speed and feed rate both 300 mm/min, set nozzle height 0.4 mm, and raster gap 1.6 mm. Figure 5b shows a picture of the ‘as printed’ square specimen and a detail of the corner region. The printed rasters are well laid following the defined printing path, but some interruption of the feed resulted in empty areas, even if the printing was continued at almost the same point as the stopping position. The linear section to produce the tensile specimen is straight following the path, but a perfect sharp 90° turn could not be achieved at the corners as the poor bed adhesion of the filament to the printing bed makes the sudden change of direction problematic [25]. The impregnated fibre presented on the surface is the result of high fibre volume fraction and imperfect fibre distribution. There are some fuzzy fibres that deviated from the printing path because of some fibre movement under the compaction nozzle.

Inspired by the post-consolidation of 3D printed continuous carbon fibre [26] that provides mechanical properties improvement by the elimination of the voids, a group of samples was separated and then thermally consolidated under a vacuum pressure (1 bar) in an oven at 180 °C for 1 h, called ‘print + oven’, to enhance the raster fusion and compare the improvement of the post-printing consolidation to the original printed part and explore the mechanical performance limitation of the material. The post-printing consolidated parts show smoother surfaces caused by the fusion of the rasters and accumulations of material on the edges, as can be seen in the comparison between the high magnification images of Figure 5c,d. Since the filament was already compacted by the modified nozzle during printing, the thickness reduction due to the consolidation is relatively small, around 10%, and caused mainly by the removal of microvoids.

To produce a uniaxial 10 mm × 100 mm tensile specimen from both ‘as printed’ and ‘print + oven’, their corners were discarded to avoid inconsistency of the printing by cutting at the transition from flat to a curved path as shown by the cutting line in Figure 5b, with a metal hand saw to make four tensile specimens. Finally, 20 mm fibreglass end tabs were attached at both ends of the 10 mm × 100 mm specimens, leaving a 60 mm gauge length. There were five large square printed parts, so a total of 20 specimens were fabricated for the as-printed testing, while there were two large square post-printing consolidated parts resulting in eight specimens of the consolidated one.

### 3.2. Tensile Testing and Result

**Figure 6:**
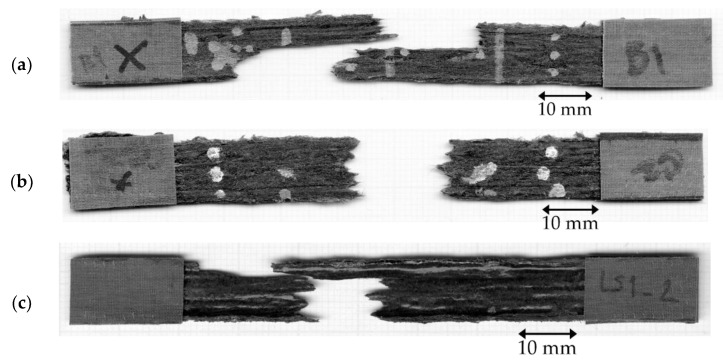
Breakage sample of tensile sample: (**a**) inter-raster failure parallel to the load direction; (**b**) favourable failure perpendicular to the load direction showing the load transfer to the fibre direction; (**c**) breakage of the oven-treated sample showing the breakage parallel to the raster direction.

A servo-electric tensile testing machine (Shimadzu, Japan) with a 1 kN load cell operated at a crosshead displacement speed of 1 mm/min was used. The strain was measured using a video extensometer (Imetrum, UK). The failure of the samples was recorded. In Table 2, the current DcAFF printed part, ‘as printed’ and ‘print + oven’ are compared to the previous HiPerDiF-PLA studies: (a) the properties of the part fabricated by the manual mould HiPerDiF-PLA filament in the previous publication by Krajangsawasdi et al. [18] and (b) HiPerDiF-PLA thin tape obtained from the consolidation machine [17]. The tensile properties of the current part are significantly increased from the previous 3D printed part, with the manually moulded HiPerDiF-PLA filament. The mechanical properties improvement from the previous printing is the result of a two-fold increase in fibre content. When comparing the DcAFF to the thin tape HiPerDiF-PLA format obtained from the consolidation machine [17], the DcAFF part presents a comparable tensile stiffness, while the strength is significantly lower. The difference in the tensile strength between the tape and printed formats is attributable to an undesirable failure mode triggered by poor raster-to-raster fusion in the printed part, resulting in an initial breakage parallel to the load direction (Figure 6a). The better load-bearing ability correlates with the localised fibre rupture rather than the delamination failure mode manifested in the breakage perpendicular to the loading direction (Figure 6b). Post-printing consolidation improves tensile properties, especially tensile stiffness. This may be associated with a reduction of out-of-plane deviation in fibre orientation. However, there is no statistically significant change in the tensile strength. This is because the inter-raster bonding is still the weak point in the sample as demonstrated by failure similar to the ‘as printed’ specimens, parallel to the printed raster, in Figure 6c. However, post-printing consolidation with heat and pressure could still be considered as an option to improve the physical and mechanical properties of the HiPerDiF-PLA after printing for simple shape parts without internal cavities.

**Figure 7:**
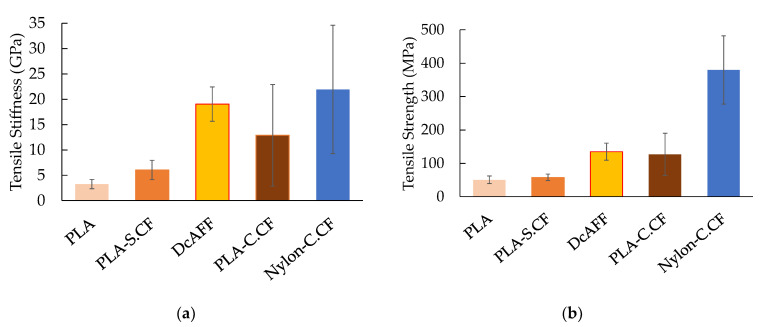
Comparison of (**a**) tensile stiffness; (**b**) tensile strength between the DcAFF (HiPerDiF-PLA) printed as a single layer 3D printed part, 20 tested specimens, and other composite 3D printings from publications: PLA [5,7,27,28,29,30,31,32,33], PLA-short carbon fibre (PLA-S.CF) [6,27,30,31,32,34,35,36,37], PLA-continuous carbon fibre (PLA-C.CF) [38,39,40,41,42], and Markforged continuous carbon fibre (nylon-C.CF) [6,14,43,44,45,46,47,48].

To compare the performance of the DcAFF (HiPerDiF-PLA) 3D printed part with other available 3D printing materials from literature, the tensile stiffness and strength of the DcAFF printed in a single layer were plotted against other PLA [5,7,27,28,29,30,31,32,33], PLA-short carbon fibre (PLA-S.CF) [6,27,30,31,32,34,35,36,37], PLA-continuous carbon fibre (PLA-C.CF) [38,39,40,41,42], and Markforged continuous carbon fibre (nylon-C.CF) [6,14,43,44,45,46,47,48]. Both plots, Figure 7, show that the properties of the DcAFF are superior to short-PLA composite 3D printed parts and at a comparable level with the PLA-continuous carbon fibres. The properties of DcAFF are still lower than commercial composite 3D printed materials, Nylon-continuous carbon fibre, produced by Markforged: the strength of Markforged is about three-fold greater than the DcAFF one. This may be due to a better fibre-matrix integration that leads to a stronger interface and better load transferability and/or the undeniably slightly higher material performance of the nylon matrix compared to the PLA. Although the strength of HiPerDiF-PLA is clearly inferior to the Markforged product, its stiffness is almost comparable. Further work needs to examine a similar nylon matrix in the same manufacturing process to make a more conclusive assessment of the matrix role.

## 4. Open-Hole Testing

This section demonstrates the benefit of bulking the tape into the fine-diameter filament for 3D printing compared to the conventional tape layup by fabricating a tensile test sample with an open hole manufactured by different methods.

### 4.1. Open-Hole Specimen Fabrication

To illustrate the benefit of using the HiPerDiF as a 3D printing material, open hole samples were fabricated with two main different methods: 3D printing with a curvilinear path and hand layup unidirectional tapes with hole punching. Two different curvilinear deposition paths were considered. One set of printed specimens was consolidated under vacuum pressure in an oven to replicate the tape layup process with the heat-compaction process. In total, four sets of specimens were tested: 10-curvature printing (10C), 4-curvature printing (4C), 4-curvature printing with post-printing consolidation (4C-Oven), and tape hand layup with heat-compaction forming (tape).

#### 4.1.1. Open-Hole Printing

**Figure 8:**
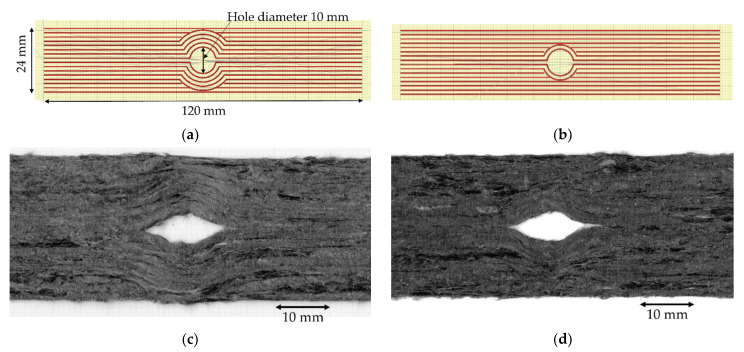
(**a**,**b**) Designed curvilinear 3D printing path for 10- and 4-curvature samples; (**c**,**d**) high magnification at the curvature hole of 10- and 4-curvature samples on the bottom side (sticking to the printing bed) [25].

To study the load-bearing behaviour of the different printing paths, the G-code was set to print the curvilinear specimen in two different ways: (10C) steering 10 rasters around the hole and keeping one straight-continuous line on each edge, Figure 8a [25]; and (4C) steering only four rasters around the hole and keeping eight straight-continuous linear lines, Figure 8b. These paths were deposited in a single layer with the same printing condition as the large square tensile specimen mentioned in the previous section. The finished parts of the 10C and 4C paths are shown in Figure 8c,d, respectively. The hole is defined by the printing path as perfectly circular with a diameter of 10 mm; however, this cannot be achieved due to the poor adhesion between the printing bed and the heated raster. At the sudden turning point from the linear to curvature, the raster cannot be held on the bed while the nozzle moves upwards and sideways. This motion drags the filament producing an eye-shaped hole rather than the one defined by the desired half-circular path. The measured hole dimension, in the direction perpendicular to the load, is around 5–6 mm.

#### 4.1.2. Post-Printing Consolidation of the Printed Part

**Figure 9:**
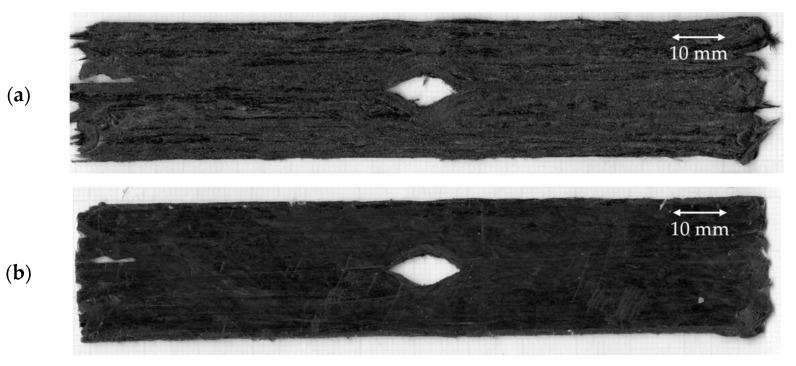
(**a**) 4-curvature printed part before post-printing consolidation; (**b**) the sample after the consolidation under heat and pressure (4C-Oven) showing a smoother surface and better raster fusion.

For a further study inspired by the thermal consolidation process carried out in the tensile testing, some of the 4C printed parts from Section 4.1.1 were consolidated in a vacuum bag (−1 bar) at a temperature of 180 °C for 1 h; those specimens are identified as 4C-Oven. This aimed at obtaining better raster bonding and reaching a performance similar to the oven-treated layup, described below in Section 4.1.3. To prevent the shape-changing of the printed part, especially the hole, cork tape was used as a dam around the specimen and inside the hole. The changes in the sample surface between the printed and the consolidated specimens with a smoother surface and well-joined rasters can be seen in Figure 9a,b. The hole shape does not change from the previous printed part, but there is material accumulation on the hole and sample edges, as the matrix cannot flow over the cork, leading to thicker edges. The overall raster direction does not change with the post-printing consolidation. The thickness reduction after the consolidation is approximately 20%, from 0.60 ± 0.043 to 0.48 ± 0.023 mm.

#### 4.1.3. Open-Hole Layup

**Figure 10:**
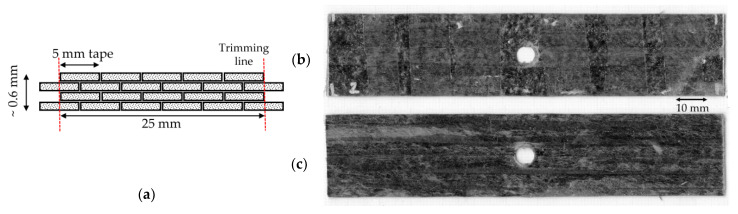
(**a**) Stacking sequence of the layup sample showing four layers with trimming lines to make a 25 mm wide sample; an example of a layup part with a cutting hole using a hollow punching tool: (**b**) top surface and (**c**) bottom surface (attached to the metallic mould) [25].

Open-hole layup samples were built by placing 5-mm-wide HiPerDiF tapes side-by-side and staggered through the thickness to obtain the desired width and thickness. The tape used in this stage is the same production batch as of the filament in the previous section, so the fibre volume fraction is in a similar range. In this case, there are four layers of tape as the thickness of the original tape is between 0.1 and 0.2 mm and the aim was to achieve a total thickness of ~0.6 mm. Figure 10a shows the cross section of the stacking sequence with the half-width overlap of six and five tapes on each layer [25]. The produced laminate was compressed and heated using the same pressure, temperature, and duration of the post-printing consolidation of the printed one as described in the previous section to ensure the fusion of the PLA composite. The achieved consolidated thickness is between 0.6 and 0.7 mm. The layup edges were trimmed with a knife, where the first and third layers protruding from the other layers, to achieve a 25-mm-wide sample. Finally, the hole was cut with a 6-mm-diameter hollow punching tool with a hammer. The punching is expected to produce a straight cut hole rather than a spinning cutting of the drill as the specimen is a thin panel. The hole size was selected to be equivalent to the size of the holes of the printed samples measured perpendicularly to the longitudinal direction. The top and bottom surfaces of the obtained specimens are shown in Figure 10b,c, respectively. The bottom surface is smoother than the top surface, as it is an imprint of the tool in which the composite is laid-up; however, some dry areas, corresponding to dry patches in the composite tapes, can be observed. The measured thickness of the layup after the oven processing is 0.7 ± 0.046 mm.

### 4.2. Open-Hole Testing Result

The samples’ width and thickness at the position of the hole were measured for stress calculation. The hole size was measured from the scanned image of the sample at the widest position of the hole. The cross-section area used to calculate the strength is the specimen width minus the hole diameter. The samples were tested using the same testing machine and procedure as the tensile tests described above. The strain was measured via digital image correlation (DIC) to obtain the strain map during loading. The DIC parameters are illustrated in Table 3.

**Figure 11:**
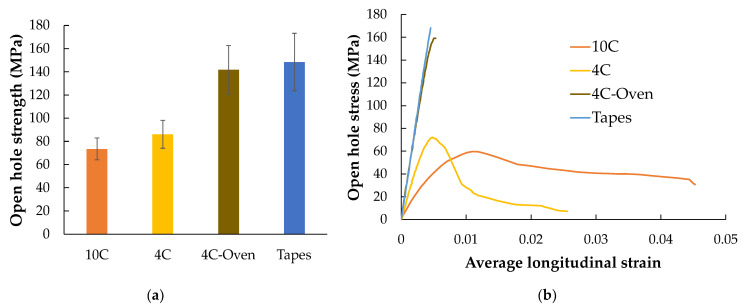
(**a**) Open-hole strength of different sample types; (**b**) open-hole stress *versus* average longitudinal strain curves of each sample type showing different failure behaviours.

**Figure 12:**
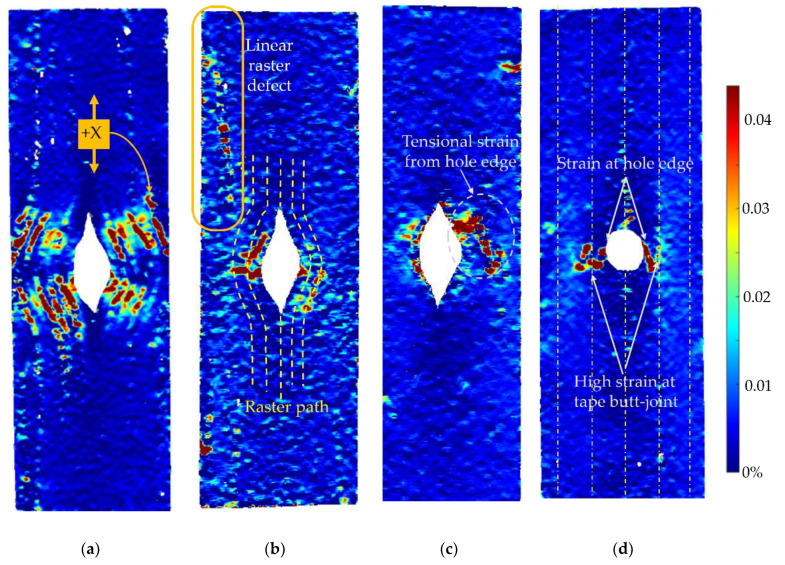
DIC analysis of longitudinal strain (ε_x_) at the maximum load of each sample in different types: (**a**) printed 10C at 59.77 MPa; (**b**) 4C at 100.5 MPa; (**c**) 4C-Oven at 159.2 MPa (post-printing consolidated part); (**d**) tapes at 166.4 MPa (layup part with a punched hole).

**Figure 13:**
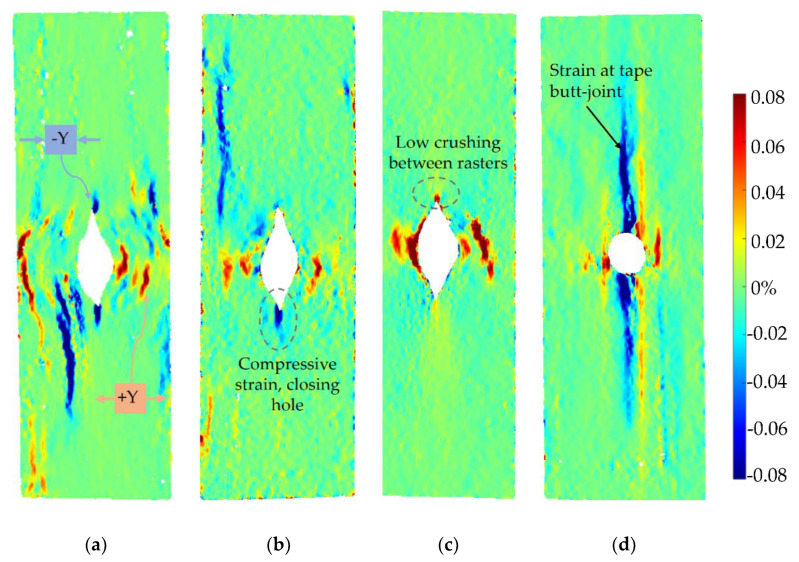
DIC analysis of transverse strain (ε_y_) at the maximum load of each sample in different types: (**a**) printed 10C at 59.77 MPa; (**b**) 4C at 100.5 MPa; (**c**) 4C-Oven at 159.2 MPa (post-printing consolidated part); (**d**) tapes at 166.4 MPa (layup part with a punched hole).

**Figure 14:**
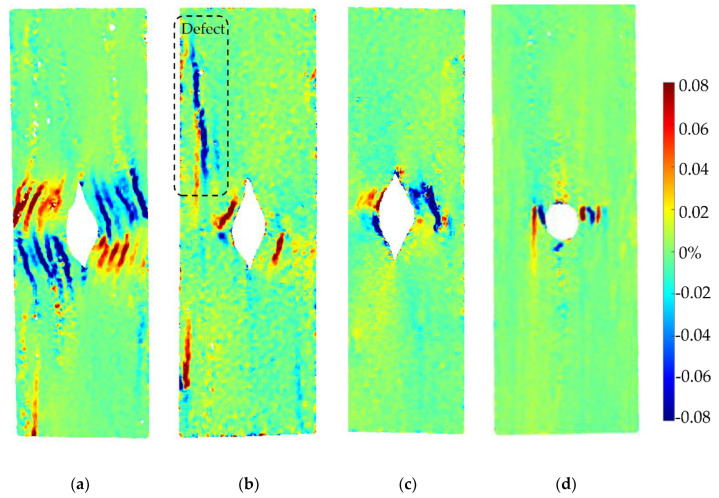
DIC analysis of in-plain shear strain (ε_xy_) at the maximum load of each sample in different types: (**a**) printed 10C at 59.77 MPa; (**b**) 4C at 100.5 MPa; (**c**) 4C-Oven at 159.2 MPa (post-printing consolidated part); (**d**) tapes at 166.4 MPa (layup part with a punched hole).

The strengths of each sample group are shown in Figure 11a. There is a significant open hole strength difference between the only printed parts (10C and 4C) and the oven-treated part (both 4C-Oven and tape layup). The oven consolidation strengthens the inter-raster bonding. The stress–strain curve of a sample from each tested group is shown in Figure 11b. This also shows the different mechanical behaviours between the post-printing consolidated parts, which show a linear behaviour with a sudden failure of the whole structure, and the ‘as printed’ specimens, which display a highly non-linear behaviour with a gradual load drop after the initial failure caused by the separation between the curvilinear rasters and the remaining structure still carry the further load. The strain maps obtained at the highest load in each sample, marked in Figure 11b, in longitudinal, transverse, and in-plane shear achieved from DIC are plotted in Figure 12, Figure 13 and Figure 14, respectively.

**Figure 15:**
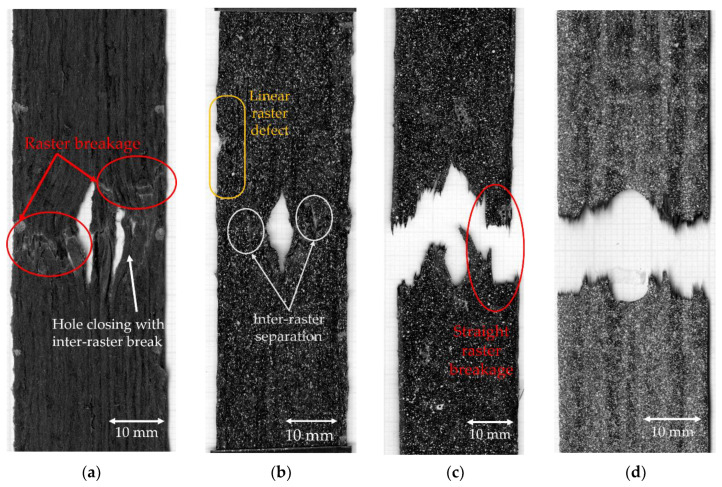
Breakage of the open-hole samples: (**a**) 10C showing hole closing by the inter-raster separation following raster breakage [25]; (**b**) 4C showing the raster separations with defect at the linear line that occurs before the breakage of the raster at the curvature area; (**c**) 4C-Oven showing the catastrophic failure in the rasters perpendicular to the load direction; (**d**) tape layup showing breakage perpendicular to the fibre/load direction at the middle of the hole [25].

As shown in Figure 11a, 10C samples show the lowest average open hole strength. This is because it has the greatest number of curvilinear rasters, which have a lower load-bearing capacity than linear rasters. It also results in the highest non-linear behaviour and shows a large deformation at failure, as seen in Figure 11b. The longitudinal strain map of the 10C (Figure 12a) shows that the tensile strain is concentrated in the inter-raster region around the curvilinear area. The tensile load in the longitudinal direction converts into compression in the transverse strain, which is shown as a high compressive strain near the sharp corner of the elliptical shape (Figure 13a). The majority strain of 10C is the shear strain (Figure 14a), which tends to separate the joint between rasters in correspondence with the curvilinear path in the 45° direction, which is weaker than the longitudinal raster direction. After the initial inter-raster failure, the printed rasters still stay connected; as seen in Figure 15a, this may benefit some applications that require a non-linear behaviour rather than a high strength with a catastrophic failure.

The 4C sample has a lower number of curvilinear rasters than 10C, which, in turn, means a greater number of linear rasters. This leads to higher open hole strength (Figure 11a) and lower non-linearity than 10C, as seen in Figure 11b, as the stress drops significantly after the maximum load. According to the strain map, the strain also accumulates around the curvilinear rasters, showing the separation between them. The strain map of a 4C sample in all directions (Figure 12b, Figure 13b and Figure 14b) also shows the high strain at the edge of the sample in correspondence to the linear rasters. This looks greater than the longitudinal strain near the curvature (Figure 12b). This may be a result of a printing defect. This leads to a weak point in the linear raster and a failure at the edge, as seen in Figure 15b, rather than only curvilinear raster separations.

After the consolidation, the 4C-Oven samples have a significant increase in the open hole strength from around 80 MPa to 140 MPa (Figure 11a). This is a similar level to the conventional tape layup sample. However, the printed part with the consolidation still shows a small non-linearity and a small plateau before the failure (Figure 11b). Thanks to the welding of the rasters due to consolidation, the inter-raster separation is clearly reduced. The initial strain concentration moves from the inter-raster around the curvilinear area to the area near the edge of the hole and the fracture starts from this point before breaking the whole sample across the hole area (Figure 15c). The separation of the rasters at the sharp corner of the eye shape has disappeared, showing a compressive strain at the sharp corner (Figure 13c) rather than the tensile strain between rasters in the printed 4C. However, there is a strain concentration at the inter-raster bonding, as clearly seen in Figure 13c, as this is one of the weakest points, but this point also offers the non-linear behaviour as mentioned above.

The tape layup with heat-compaction forming shows the highest open hole strength in this experiment (Figure 11a). The strength is at a similar level to the 4C-Oven one, this confirms that the consolidation strengthens the raster bonding. Moreover, there is no non-linearity in this sample, as seen in Figure 11b. The sample suddenly fails after the maximum load is reached, as seen in Figure 15d. Instead of symmetric strain distribution around the hole, there are several small high strain spots around the hole edges, as shown in Figure 12d. This may be because of the different behaviour of the discontinuous fibre and the imperfect bonding between tapes as shown by the strain concentration as the vertical lines parallel to the tape edges, as seen in Figure 13d. The latter reason can be emphasized by the strain concentration at the butting lines between the tapes, clearly seen in Figure 13d. The high compressive strain in the transverse direction results from the butting line between two tapes that move against each other.

## 5. Printing Precision Improvement

### 5.1. Sharp Corner Angle

According to Figure 5b, the printed raster does not follow the turning 90° corner. The reason for the discrepancy between the intended and actual fibre paths is that the finite stiffness of the filaments results in considerable in-plane bending stresses around the tight corners, which overcome the adhesion with the substrate. Obviously, the tighter the radius of curvature, the greater the bending stresses and the bigger the challenge is to steer as intended. An additional factor is the point of pressure application of the print head, which is offset from the extrusion axes and, hence, offsets the point where filaments can be subjected to consolidation pressure by the nozzle.

**Figure 16:**
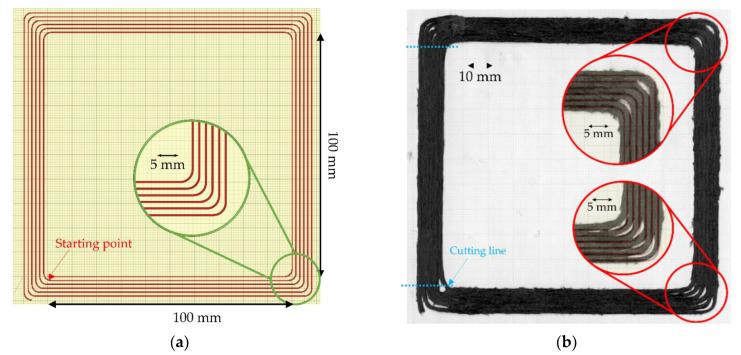
(**a**) Printing path of large square spiral with 3-mm turning radius at the corners; (**b**) top surface of the radius printed part with a zoom-in of a corner.

To minimise the tight angle turning, another smooth turning printing path with a radius of 3 mm was devised to deal with the sudden change in the printing direction. The printing path was designed as shown in Figure 16a, and the actual printing is shown in Figure 16b. These smoother curve corners progressively change the printing direction and show the steering efficiency of the material in 3D printing. Looking at the corners of the printed part with high magnification (Figure 16b), it can be observed that, even if some printed rasters followed the defined printing path, there are some deviations caused, as above, by the poor adhesion to the printing bed. Further investigation is required to identify the optimal steering corner radius; however, from the empirical trials above it can be suggested that the practical limit is 3 mm.

### 5.2. Curvilinear for Open-Hole Sample

**Figure 17:**
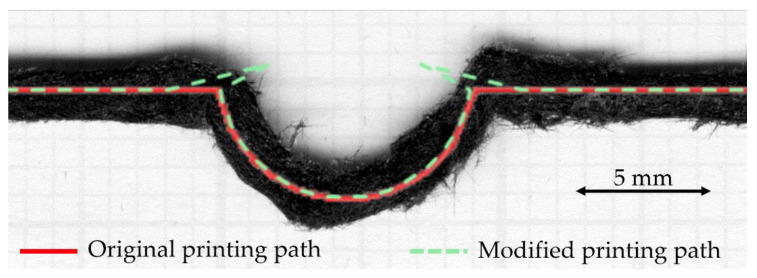
Curvilinear printing for open hole sample with a compensated printing path by moving further inward to the circular section before leaving back to the defined path.

As can be seen from the open hole curvilinear printed sample, the holes are elliptically shaped rather than a circular shape as the predefined printing path. This is expected to be the result of (a) poor bed adhesion that cannot pin the raster before entering the circle section; and (b) the nozzle design that has a filleted end, so the raster is not fully compressed at the turning position. To close the separation of the raster at the changing of linear to curvature, an additional path, as shown in Figure 17, that goes inwards to the circle more than the defined path was introduced. The action produces a sharper corner and offers higher time to build adhesion to the printing bed. The circular curve is slightly offset from the defined path, but the overall dimension almost complies with the defined path. Further work on redesigning the print path to match the nozzle features and adhesion characteristics is needed to establish an optimal compensation path and to improve the print precision.

## 6. Conclusions

In this study, the transformation of ADFRC tapes produced with the HiPerDiF technology, and a PLA matrix, into circular cross-section filaments suitable for 3D printing have been developed from the previous labour- and time-intensive manual moulding method to a newly developed and automated process that offers a higher production rate. The filament, now called DcAFF, has been used to 3D print open-hole samples to investigate the manufacturing and mechanical performance of such a material. The key findings and the potential future works can be summarised as follows:

- The application of the HiPerDiF thin flat tape was expanded from tape layup to 3D printing by turning the tape into a circular cross section filament using an industrially scalable method developed ad-hoc. The fibre volume content of the filament is around 21–25%, which is relatively high compared to other 3D printing composite filaments. However, some defects, e.g., microvoids, in the filament could be reduced by tuning the process parameters, i.e., temperature, speed, and roller gap, to increase the performance of the filament. This could be considered in future work.
- The tensile performance of the filament was evaluated by printing the produced filament into a single-layer specimen. The printed part shows higher tensile stiffness and strength than other PLA 3D-printed composites. This shows the potential of the produced filament to be used as any commercial filament. To improve the quality of the printed part, the 3D printing parameters, e.g., printing speed and raster gap, should be studied and/or optimized. In addition, thermal annealing with pressure can also enhance the properties of the part.
- The benefits of fabricating the HiPerDiF tape into circular cross-section 3D printing filament were emphasized by making an open-hole specimen for the tensile testing. The 3D printing part reduces the layup labour, time, and required skills. The curvilinear open hole 3D printed sample showed different stress development under tensile load from the stress concentration at the hole edges to the inter-raster separation. This resulted in a change of failure mechanism from brittle breakage that occurred in the layup with thermal annealing to the more progressive failure mode by breaking at the inter-raster bonding around the curvature. After the initial inter-raster failure, the rasters still stay connected for a while before its completed failure. This will be beneficial to applications that require non-catastrophic failure.
- Although the open hole sample fabricated with 3D printing to the curvilinear printing path shows lower strength than the layup because of the better plastic fusion under the heat-compaction process, the 3D printed curvilinear part with the post-printing consolidation shows relatively similar strength to the laid-up one. This demonstrates that the 3D printed filament and the tape could have similar performances when fabricated with the same method.
- The steering of the filament along the curve path is possible even if the deposition resolution/accuracy is now relatively low due to the poor raster-bed adhesion. This may be caused by the high fibre volume content and the consequently low amount of thermoplastic available to promote adhesion to the bed. Moreover, the stiffness of the material, long fibre and high viscosity thermoplastic, results in the in-plane bending during turning that overcomes the bed adhesion. A tiny radius of 3 mm is recommended to replace the sharp 90° corner. For the curvilinear path, the proper path compensation should be introduced to provide a good turning from linear to entering the curvature.

## Figures and Tables

**Table 1:** Raw material, PLA and fibre, properties.

	Density (g/cm^3^)	Tensile Stiffness (MPa)	Tensile Strength (MPa)
PLA [21]	1.24	3861	144
Fibre [19]	1.82	225 × 10^3^	4344

**Table 2:** Tensile properties comparison of the current DcAFF samples to the previous studies.

Specimen Format	Fibre Volume Fraction	Tensile Stiffness (GPa)	Tensile Strength (MPa)
DcAFF “As printed”	21–25%	19 ± 3	132 ± 21
DcAFF “Print + oven”	21–25%	24 ± 2	151 ± 29
Tape [17]	12–13%	24 ± 4	274 ± 31
Single layer (manual) [18]	10–18%	14 ± 2	104 ± 32

**Table 3:** DIC technique parameters.

**Software**	Davis10.1.2	**Image resolution**	2056 × 2418 pixel
**Camera and Lens**	M-lite & 35 mm	**Field of view**	78.95 mm × 95.27 mm
**Correlation mode**	Sum of Difference	**Frame rate**	1 image per second
**Subset size**	21 × 21 pixel (0.8 mm × 0.8 mm)	**Strain resolution**	7.56 × 10^−4^ ε
**Step size**	2 pixel (0.076 mm)	**Scale factor**	26.04 pixel/mm

## Data Availability

All data required for reproducibility are provided within the paper.
